# Enhancing Cycle Stability in LiNiO_2_ with Phase transition suppression via Crystalline Disordered Surface Layer

**DOI:** 10.1002/advs.202503476

**Published:** 2025-07-18

**Authors:** Sooyeon Choi, Dong‐hee Lee, Yonghyeon Kwon, Maxim Avdeev, Seok Hyun Song, Minki Kim, Sehyun Kim, Seung‐yong Lee, Hyungsub Kim, Minkyung Kim

**Affiliations:** ^1^ Department of Electronic Materials Engineering Kwangwoon University 60 Gwangun‐ro 1‐gil, Nowon‐gu Seoul 01897 Republic of Korea; ^2^ Australian Nuclear Science and Technology Organisation New Illawarra Road, Lucas Heights Sydney New South Wales 2234 Australia; ^3^ School of Chemistry The University of Sydney Sydney 2006 Australia; ^4^ Neutron Science Division Korea Atomic Energy Research Institute (KAERI) 111 Daedeok‐daero 989 Beon‐Gil, Yuseong‐gu Daejeon 34057 Republic of Korea; ^5^ Gwangju Clean Energy Research Center Korea Institute of Energy Research (KIER) 270‐25 Samso‐ro, Buk‐gu Gwangju 61003 Republic of Korea; ^6^ Division of Materials Science and Engineering Hanyang University Seoul 04763 Republic of Korea; ^7^ Department of Battery Engineering Hanyang University Seoul 04763 Republic of Korea; ^8^ Advanced Energy Research Institute Chungbuk National University Cheongju Chungbuk 28644 Republic of Korea

**Keywords:** cycle stability, Li/Ni disordered phase, Ni‐rich lithium layered oxides, phase transition suppression, surface modification

## Abstract

Ni‐rich layered oxides are promising cathode materials for lithium‐ion batteries but require improved stability to meet performance demands. To address this, doping and surface modification are commonly employed stabilization strategies, each offering distinct benefits. Doping primarily suppresses phase transitions, thereby reducing volume changes, while surface coatings protect the material by minimizing continuous reactions with electrolytes during cycling. Consequently, these approaches are often used in combination to enhance material stability. This study demonstrates that a crystalline disordered surface layer can effectively suppress structural changes without doping. Notably, the thickness of the disordered surface layer is successfully controlled during synthesis for the first time. The results reveal that the formation rate of the layered structure plays a critical role in controlling the rock‐salt disordered surface layer. Although a thicker crystalline disordered surface layer in single‐crystal LiNiO_2_ resulted in a slight capacity reduction, it exhibits significantly improved capacity retention, maintaining up to 84% of its capacity after 500 cycles in a full‐cell test, while also substantially reducing voltage decay. This study provides insights, demonstrating that well‐controlled surface modification can simultaneously protect the surface and mitigate structural changes, paving the way for the development of stable cathode materials.

## Graphical abstract

The thickness of the disordered surface phase on single‐crystal LiNiO_2_ is controlled during synthesis. LiNiO_2_ with a crystalline disordered surface layer exhibits suppression of the H2–H3 phase transition. This mitigates structural degradation with improved cycling stability. The findings highlight that a crystalline surface layer can regulate bulk structural behavior, similar to the effects of doping.

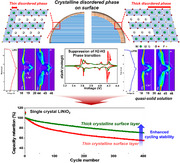

## 1 Introduction

Lithium‐ion batteries (LIBs) have attracted significant attention due to their widespread applications in grid storage systems, portable electronics, and electric vehicles. In response to increasing demand, extensive research efforts have focused on improving cathode materials that offer both high energy density and low cost. One of the most prominent trends in this research is the reduction, or ideally elimination, of cobalt in favor of higher nickel content within lithium layered oxide structures, such as LiNi_x_Co_y_Al_1−x−y_O_2_ (NCA) and LiNi_x_Co_y_Mn_1−x−y_O_2_ (NCM) cathodes, where nickel concentrations exceed 80%.^[^ ^1^ ^]^ Cathodes with over 90% nickel content will soon launch on the market. As a result, LiNiO_2_ is emerging as a target material for further development in next‐generation LIBs.

However, increasing the nickel content in lithium layered oxides presents several challenges. First, Ni^3^⁺ is thermodynamically not as stable as Ni^2^⁺ in the structure and tends to reduce to Ni^2^⁺.^[^ ^2^ ^]^ This instability causes Ni‐rich lithium layered oxides to lose lithium ions from the structure to the surface as Ni^3^⁺ is reduced to Ni^2^⁺. The lithium on the surface is likely to react with moisture and CO_2_ in the air, which resulting in the formation of residual lithium compounds such as Li_2_CO_3_ and LiOH on the surface.^[^ ^3^ ^]^ They cause slurry gelation and gas evolution during battery operation that degrade materials. Moreover, the reduction of Ni^3^⁺ to Ni^2^⁺ increases the likelihood of cation mixing defect, where Ni^2^⁺ ions occupy lithium sites.^[^ ^4^ ^]^ This defect significantly impedes lithium‐ion diffusion, which negatively impacts the electrochemical performance.^[^ ^4^, ^5^ ^]^ Not only chemical instability but also structural instability becomes rise with increasing nickel content. The materials experience more pronounced volume changes during charge and discharge.^[^ ^6^ ^]^ Specifically, the contraction of the *c* parameter during the H2‐H3 phase transition is more significant compared to the relatively smaller decrease in the *a* parameter. This anisotropic volume change induces strain and stress within the particles, leading to interparticle cracks, which degrades capacity retention rapidly.^[^ ^7^ ^]^

To address these challenges, a general approach is doping with various elements in the Ni‐rich cathode.^[^ ^8^ ^]^ Recently, it is revealed that doping results in two different effects. Dopants such as Mg^2+^, Al^3+^, Zr^4+,^ and Ti^4+^ generally stabilize the structure, which suppress the phase transition on (dis)charging. They also suppress Ni migration into lithium sites by increasing the thermodynamic barrier. On the other hand, high valence elements such as Nb^5+^, Ta^5+^, Mo^6+^, and W^6+^ influence the particle morphology.^[^ ^9^ ^]^ They inhibit grain coarsening at the grain boundary and maximize the crystallinity of Ni‐rich layered oxides. The radially aligned primary particle morphology helps alleviate stress caused by anisotropic volume changes during cycling, which in turn reduces particle cracking and enhances cycling stability.^[^ ^9a^ ^]^ While doping is an effective strategy to stabilize bulk structure, they do not adequately address surface degradation. Surface degradation is a major contributor to overall performance degradation during cycling, as the surface is in continuous contact with the electrolyte and is thus more prone to chemical instability and degradation.^[^ ^10^ ^]^ To mitigate surface degradation, surface coatings and concentration‐gradient cathodes have been proposed.^[^ ^11^ ^]^ These strategies aim to protect the vulnerable surface by covering it with stable elements that enhance surface stability. While these modifications significantly improve the stability of the material, they are generally insufficient to prevent internal structural changes that occur during cycling.

In this work, structural degradation in single‐crystal LiNiO_2_ were effectively suppressed by a crystalline surface modification. The single crystal LiNiO_2_ was synthesized using molten salt synthesis during which a Li/Ni disordered phase formed as a surface coating layer by fluorine precursor without requiring any additional processing. The thickness of this disordered surface layer was controlled by adjusting the amount of LiF precursor used during synthesis. Notably, structural changes, particularly in the large volume change region (H2‐H3), were suppressed, and this suppression was reinforced as the surface layer thickness increased. As a result, cycling stability was significantly improved, and the irreversible phase transition from O3 to O1 at high voltages was reduced. It is commonly assumed that surface coatings do not influence bulk structural changes. However, this study demonstrated that a crystalline surface layer can constrain bulk volume changes, unlike amorphous coatings. These findings show that the strategic construction of a high‐crystallinity surface not only suppresses structural changes but also enhances surface stability. This approach provides a simple and effective method to stabilize both the bulk and surface of the material.

## 2 Results and Discussion

### 2.1 Disordering Phase Induced by Fluorine on the Surface of LiNiO_2_

**Figure 1:**
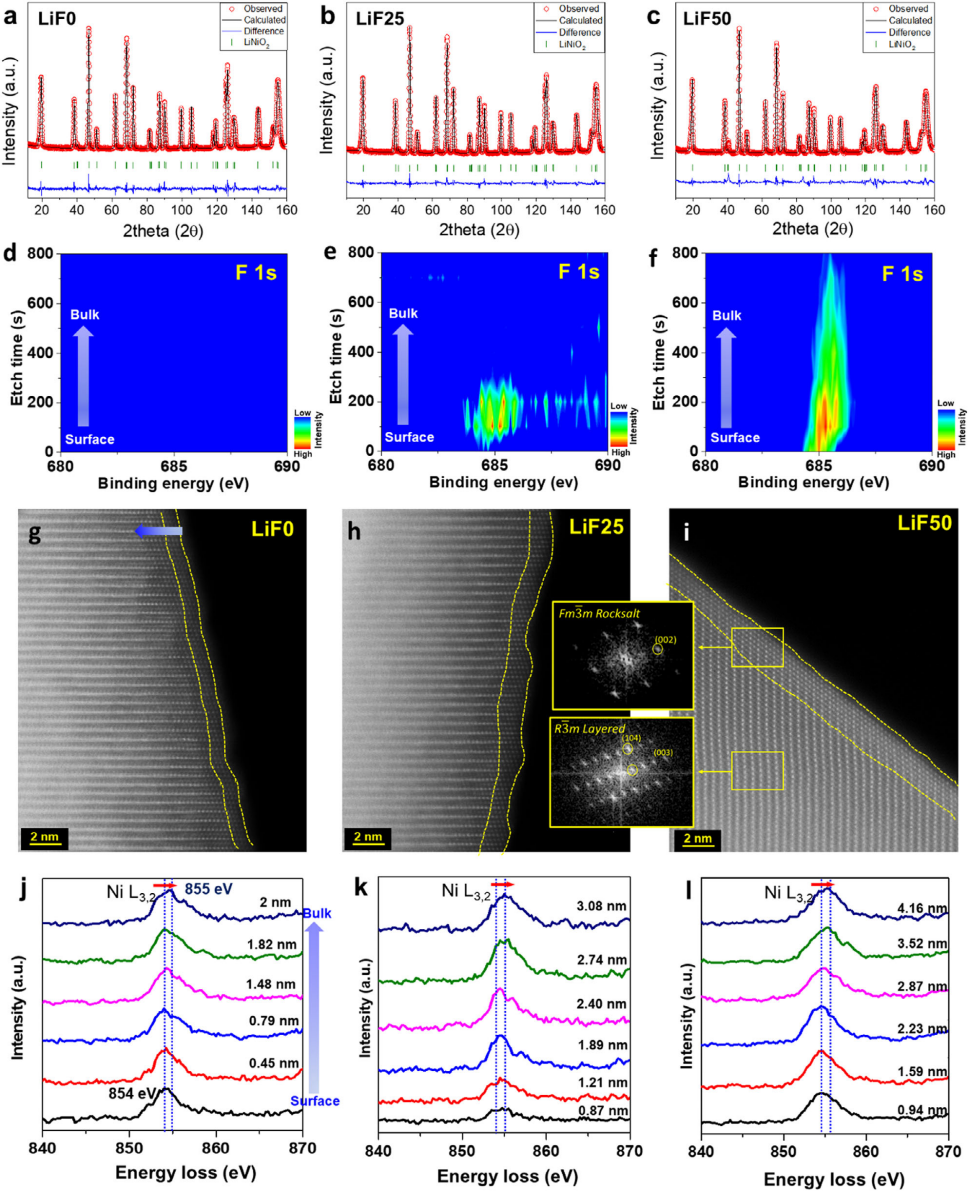
Characterization of synthesized single crystal LiNiO_2_ Neutron diffraction patterns of a) LiF0, b) LiF25, and c) LiF50. Red dots, black line, blue line, and green vertical tick marks represent the observed pattern, calculated pattern, difference between the observed and calculated patterns, and LiNiO_2_ Bragg peaks, respectively. XPS spectra of F 1s as a function of etching time (depth profile) of d) LiF0, e) LiF25, and f) LiF50. High‐angle annular dark field–scanning transmission electron microscopy images (HAADF‐STEM) and Ni L‐edge spectra of electron energy loss spectroscopy (EELS) from surface to bulk (depths are in the graphs): g,j) LiF0; h,k) LiF25; and i,l) LiF50.

Single‐crystal LiNiO_2_ was prepared using a molten salt synthesis. LiOH and Li_2_SO_4_ were used as salts, as described in our previous study.^[^ ^12^ ^]^ Two lithium precursors, lithium hydroxide (LiOH) and lithium fluoride (LiF) were utilized for the formation of LiNiO_2_. The ratio of lithium precursor was varied as follows; 1) LiOH only, 2) LiOH: LiF = 75: 25 mol%, and 3) LiOH: LiF = 50: 50 mol%. The resulting samples were named LiF0, LiF25, and LiF50 respectively. Neutron diffraction (ND) was performed to analyze the bulk structures of the samples. LiF0, LiF25, and LiF50 samples exhibit pure LiNiO_2_ without any impurity phases, as shown in **Figure** **1**a–c. However, increasing LiF contents to 75 mol% (LiF75) resulted in the formation of γ‐LiNiO_2_ (tetragonal, space group) and LiF form as impurities in Figure S1 (Supporting Information). Therefore, only the LiF0, LiF25, and LiF50 were investigated in this work. Based on Rietveld refinement of the ND patterns, Lattice parameters are *a* = 2.87559 (3) Å, *c* = 14.18749 (17) Å for LiF0, *a* = 2.87530 (3) Å, *c* = 14.18628 (21) Å for LiF25 and *a* = 2.87490 (6) Å, *c* = 14.18586 (38) Å for LiF50 respectively. As LiF content increases, both *a* and *c* parameters decrease slightly. However, since the differences are below 0.001Å, the changes are not significant. Additionally, cation mixing defects in the bulk were similar across the samples with the values of 2.9%, 2.7% and 3.3% for LiF0, LiF25, and LiF50 respectively (Tables S1 and S2, Supporting Information). Previous study^[^ ^13^ ^]^ have shown that fluorine doping into the structure increases lattice parameters due to the larger ionic radius of fluorine compared to oxygen. In this study, the calculated lattice parameters suggest that fluorine does not incorporate into the bulk structure in any of the three samples. Therefore, the bulk structures remain similar across all samples.

Surface chemistry was analyzed to investigate the differences among the three samples. X‐ray photoelectron spectroscopy (XPS) was used to examine the chemical states on the surface. As shown in the F 1s spectra in Figure S2 (Supporting Information), fluorine peaks were observed in the LiF25 and LiF50 samples but not in LiF0. As the LiF ratio increased, the fluorine peak became more pronounced, and its intensity increased. The peak position at ≈685 eV corresponds to Li─F bonds.^[^ ^14^ ^]^ This indicates that fluorine from the LiF precursor segregates on the surface rather than doping uniformly into the bulk. To confirm fluorine segregation, XPS depth profiling was conducted in Figure 1d–f. In the LiF25 sample, fluorine was detected near the surface only. In the LiF50 sample, fluorine was present in higher contents but its intensity gradually decreases toward the bulk (Figure 1f). This confirms that fluorine segregates on the surface during synthesis.

In XPS results in Figure S2 (Supporting Information), the intensity of the Ni^3+^ peak decreases with increasing LiF content, while the Ni^2^⁺ peak intensity increased. The segregated fluorine (F^‐^) on the surface replaces oxygen (O^2‐^), reducing nickel oxidation to Ni^2+^ to maintain charge neutrality. The increase in Ni^2^⁺ content suggests that disordering defects increase on the surface, as Ni^2^⁺ readily exchanges sites with Li⁺ due to their similar ionic radii. Therefore, fluorine segregation on the surface can induce an increase in the disordered phase. To confirm the surface disordering phase, surface structure was analyzed using high‐angle annular dark field–scanning transmission electron microscopy (HAADF‐STEM) in Figure 1g–i. Disordered phase was observed on the surface of all samples (yellow region), characterized by intermixed bright (Ni) and darker (Li) atoms, in contrast to the bulk structure. As shown in Figure 1i, diffraction patterns converted by fast Fourier transformation (FFT) of the STEM image exhibits rock salt phase on the surface and layered structure in the bulk. The thickness of the disordered phase varied among the samples. As the LiF ratio increased, the disordered phase on the surface became thicker, likely due to the influence of segregated fluorine on its formation.

Additionally, Ni oxidation states were examined using electron energy loss spectroscopy (EELS) in combination with STEM. The Ni L‐edge spectrum was measured from the surface to the interior of the samples in Figure 1j,k. Specific locations of EELS analysis for the sample are shown in Figure S9 (Supporting Information). The Ni L‐edge peak exhibits a distinct shift from 854 eV toward higher energy, 855 eV as the probed spot moves from the surface to the inside. The peak position of the 855 eV indicates Ni^3+^ whereas a lower peak position corresponds to a lower oxidation state of nickel (<Ni^3^⁺). In the LiF0 sample, the oxidation of Ni is less than +3 within ≈2nm from the surface as shown in Figure 1j. With the same method, the depth of the energy shift to 855 eV was found to be 3.08 and 4.16 nm for the LiF25 and LiF50 respectively, as shown in Figure 1k,l. This indicates that the disordered phase on the surface becomes slightly thicker with increasing LiF precursor content. These results demonstrate that the LiF precursor influences surface chemistry rather than the bulk, with fluorine on the surface reducing the oxidation state of nickel and leading to the formation of the disordered phase.

### 2.2 Suppressed Phase Transition by Disordering Phase on Surface

**Figure 2:**
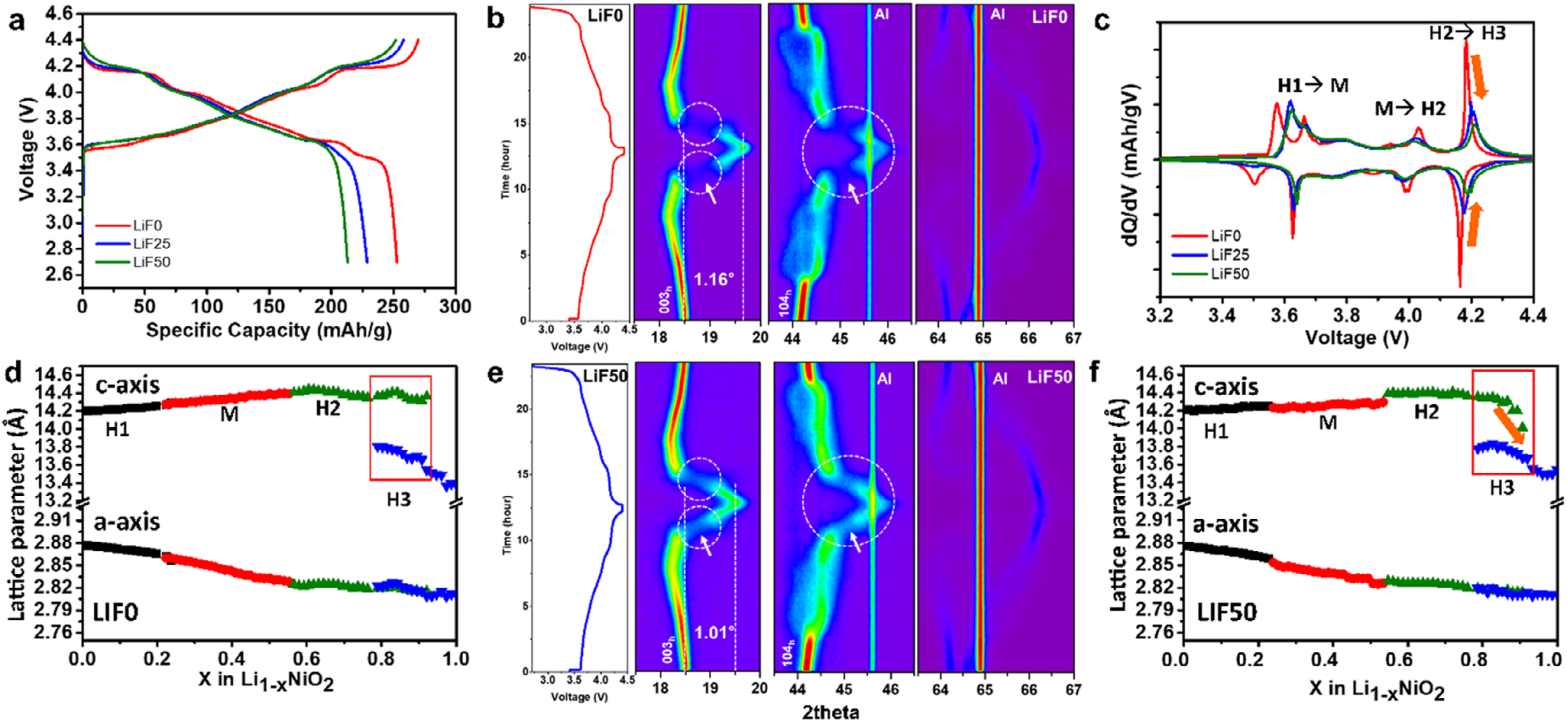
Phase transition of synthesized single crystal LiNiO_2_ a) voltage profiles of the first cycle at 0.1C c) corresponding differential capacity versus voltage (dQ/dV) plots of LiF0, LiF25, and LiF50. In situ XRD patterns of b) LiF0 and e) LiF50. Calculated lattice parameters derived from the in situ XRD patterns of d) LiF0 and f) LiF50.

The electrochemical properties of the samples were evaluated. **Figure** **2**a presents the first cycle voltage versus specific capacity profiles at C/10, within the voltage range of 2.7–4.4 V versus Li^+^/Li. Discharge capacities of 252.7, 228.6, and 212.9 mAh g^−1^ were achieved for LiF0, LiF25, and LiF50 respectively. From the galvanostatic intermittent titration technique (GITT) results in Figure S4 (Supporting Information), discharge capacities of 266, 258, and 247 mAh g^−1^ were observed, representing quasi‐thermodynamic obtainable capacities. A comparison of discharge capacities at C/10 reveals that capacity loss increases with the thickening of the disordering surface layer. Additionally, average lithium diffusivities of D_Li_ (Li_x>0.6_NiO_2_ on discharging) were calculated using GITT data (Figure S4, Supporting Information). LiF0 shows the highest average D_Li_ and becomes smaller in LiF25 and LiF50 (Figure S4h–j, Supporting Information), which coincides with the region where capacity loss is most pronounced on the discharging region. The discharge region of Li_x>0.8_NiO_2_ likely reflects a greater contribution from surface reactions compared to other regions, as it corresponds to the initial (or final) stage of the reaction. Consequently, the disordered surface layer increase resistance in the region, which in turn decreases capacity. This is due to nickel atoms blocking diffusion pathways within the disordered phase, making it unfavorable for lithium transport and ultimately reducing capacity.

To further analyze the electrochemical reactions, the differential capacity versus voltage (dQ/dV) plots of the initial voltage profiles are presented in Figure 2c. LiNiO_2_ undergoes several phase transitions. All the sample exhibits distinct phase transitions, involving the sequential changes from hexagonal (H1) to monoclinic (M), monoclinic (M) to hexagonal (H2), and hexagonal (H2) to hexagonal (H3), respectively.^[^ ^15^ ^]^ However, differences were observed between the samples. First, the initial peak during charging appears at ≈3.57 V in LiF0, whereas this peak shifts to a higher voltage of ≈3.62 V in both LiF25 and LiF50. This shift indicates that a higher activation energy is required for the reaction in LiF25 and LiF50 compared to LiF0. Unlike the first peak associated with the reaction in the H1 phase, the peaks corresponding to the H1‐M and M‐H2 phase transitions occur at similar voltages for all samples. However, the peaks in LiF25 and LiF50 are broader and exhibit lower intensity. The final peak, which represents the H2‐H3 phase transition, occurs at different voltages during charging and discharging: 4.18 V (charge)/4.16 V (discharge) for LiF0, 4.20 V/4.18 V for LiF25, and 4.21 V/4.19 V for LiF50. This demonstrates a progressive increase in voltage with increasing LiF ratios, indicating that the H2‐H3 phase transition is delayed and suppressed as the LiF ratio increases. These results suggest that the disordered phase on the surface inhibits the H2‐H3 phase transition of LiNiO_2_ during charging and discharging. Furthermore, a thicker disordered surface layer results in greater suppression of this transition.

**Figure 3:**
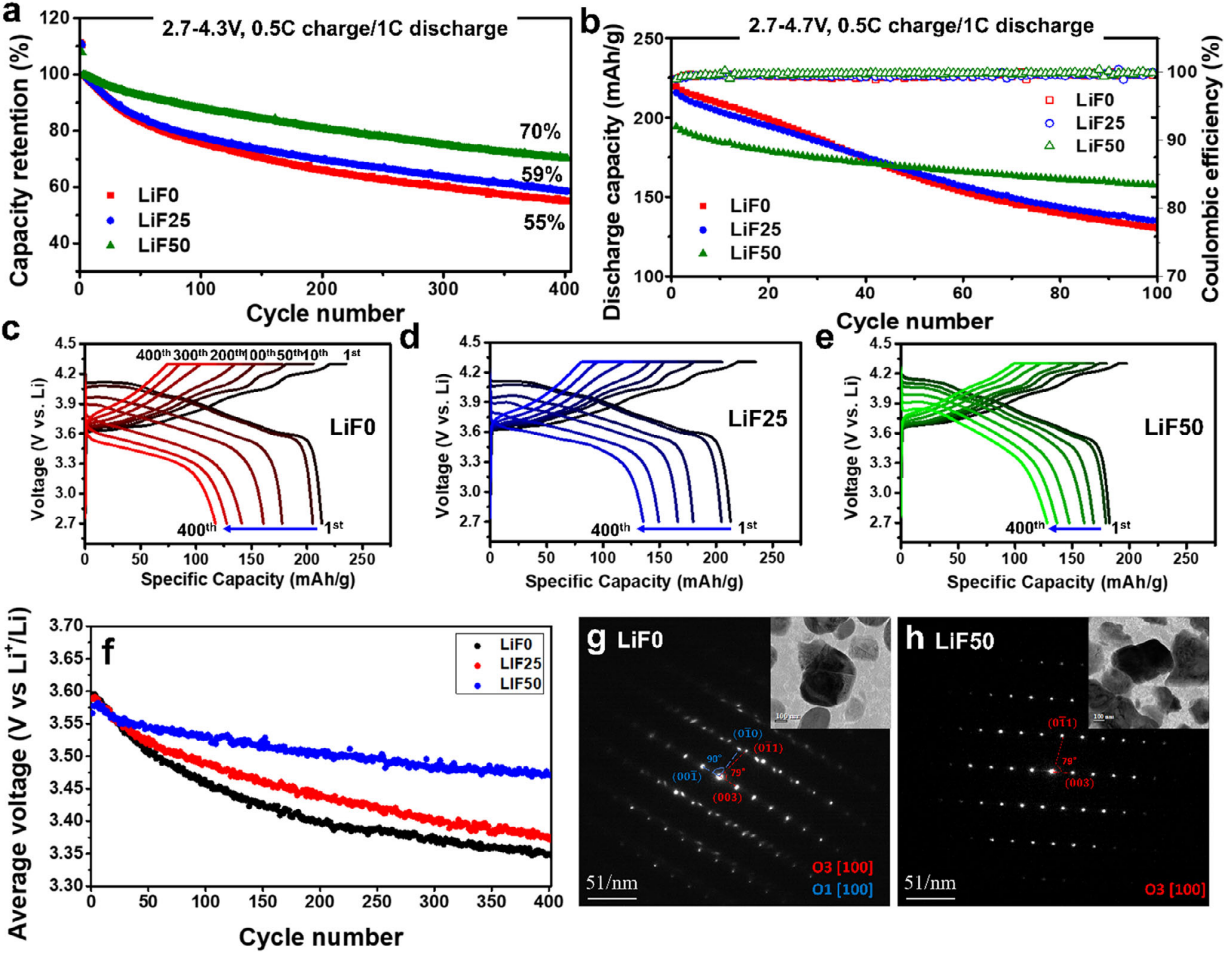
Electrochemical performance of the synthesized single crystal LiNiO_2_ a) Cycling stability with normalized capacity at charging at 0.5C and discharging at 1C of LiF0, LiF25, and LiF50 (2.7–4.3V) b) cycling stability up to 4.7 V for 100 cycles, with charging at 0.5C and discharging at 1C, for LiF0, LiF25, and LiF50. Voltage profiles on cycling (a) condition of c) LiF0, d) LiF25, and e) LiF50. f) Average discharge voltages of the cycling results of (a). Electron diffraction patterns (EDP) of charged particles investigated using transmission electron microscopy (TEM) for g) LiF0 and h) LiF50.

To directly observe the phase transition differences, in situ XRD analysis was performed on two samples, LiF0 and LiF50, as shown in **Figure** **3**c,d. At the end of charging, the (003) peak of the H3 phase appears at 19.5° in LiF50, which is a lower angle compared to the H3 peak position of LiF0 at 19.66°. When analyzing the peak shift relative to the (003) peak of the H1 phase, shifts of 1.16° and 1.01° were observed for LiF0 and LiF50, respectively. A higher 2*theta* angle for the peak indicates a significant contraction of the interlayer spacing in the H3.^[^ ^7^, ^16^ ^]^ Indeed, the lattice parameters of the H3 at the end of the charge, obtained via profile matching of the in situ XRD patterns, are 2.81134(49) Å, 13.38287(421) Å for the LiF0, 2.81104(55) Å, 13.54219(540) Å for LiF50. Notably, the *c*‐lattice parameter of the H3 phase contracts more significantly in the LiF50.

Additionally, the H2‐H3 phase transition, highlighted with circles and arrows in Figure 3b,e, shows a distinct difference between the two samples. In the LiF50 sample, the (003) peak gradually shifts from H2 to H3 during charging (and similarly during discharging), indicating a quasi‐solid solution behavior.^[^ ^17^ ^]^ In contrast, the LiF0 sample exhibits clear peak separation in this region, showing a sharper phase transition. The lattice parameters further support these findings (Figure 3d,f). For LiF50, the *c*‐lattice parameter of the H2 phase decreases to 14.00 Å, while in LiF0, it remains relatively stable at 14.36 Å. This reduction in lattice parameter mismatch between the H2 and H3 phases in LiF50 suggests that less strain and stress are generated during cycling compared to LiF0c. It is well‐established that a suppressed phase transition, particularly the H2‐H3 transition, helps to reduce volume changes, thereby improving cycle stability. Therefore, LiF50 is expected to demonstrate better cycling stability than LiF0.

### 2.3 Improved Cycle Stability by Disordering Phase on Surface

Cycling stability was evaluated, as shown in Figure 3. The samples were charged at 0.5C and discharged at 1C under constant current‐constant voltage (CCCV) charge conditions, following the initial two‐cycle formation process at 0.1C. LiF0 exhibited the highest specific discharge capacity of 213.2 mAh/g at 1C, with decreasing capacities observed for LiF25 (212.8 mAh g^−1^) and LiF50 (182.6 mAh g^−1^) as the LiF ratio increased. While discharge capacity decreased due to the slow Li diffusion and suppressed H2‐H3 phase transition, higher LiF ratio samples resulted in enhanced cycling stability, as shown in Figure 3a. After 400 cycles, 55.1%, 58.6% and 70.1% of capacity was retained for LiF0, LiF25, and LiF50 respectively. The cycling voltage profiles are depicted in Figure 3c–e, revealing that stability improved not only in terms of capacity but also voltage. For instance, at the 400th cycle, the discharge voltage for the LiF0 sample dropped below 3.6 V, whereas the LiF50 sample maintained a higher discharge voltage of ≈3.8 V. Voltage decay during cycling was mitigated with an increasing surface layer. To provide a clearer comparison, average discharge voltages are presented in Figure 3f. As a result, energy density retention, considering both capacity and voltage, was improved in the LiF50 sample.

A cycling test conducted under harsher conditions, with a high cutoff voltage of 4.7 V for 100 cycles, is shown in Figure 3b. The initial specific capacities were 219.5 mAh g^−1^ for LiF0, 215.7 mAh g^−1^ for LiF25, and 194.3 mAh g^−1^ for LiF50. After 100 cycles, the capacity retention was 59.5%, 62.7%, and 81.0% for LiF0, LiF25, and LiF50, respectively. Notably, the LiF50 sample demonstrated excellent stability under the high‐voltage cutoff condition. The corresponding voltage profiles, shown in Figure S5 (Supporting Information), reveal that the LiF50 sample maintained a high working voltage of ≈3.8 V, even under these harsh conditions. Given its superior stability, the LiF50 was further tested in a full‐cell configuration (Figure S6, Supporting Information). Although its initial discharge capacity (176 mAh g^−1^) was lower than that of LiF0 (210 mAh g^−1^ at 1C), the LiF50 sample demonstrated superior stable electrochemical performance over 500 cycles, retaining 84% of its capacity. These results confirm that a 100% Ni‐layered oxide cathode can achieve improved stability through surface disordering without requiring doping.

LiF0 and LiF50 samples were charged to 4.7 V, and their charged particles were examined using electron diffraction patterns (EDP) via transmission electron microscopy (TEM), as shown in Figure 3g,h. In LiF0, two distinct diffraction patterns corresponding to the O3 and O1 phases were observed. In contrast, LiF50 exhibited only the O3 phase. These observations were consistent across multiple particles. Most particles in the LiF0 sample showed a combination of O3 and O1 phases, whereas the majority of particles in the LiF50 sample exhibited only the O3 phase. Thus, the LiF50 sample had fewer O1 defect phases at the end of charging. These findings indicate that the disordered surface layer effectively suppresses the H3 phase transition, reducing the formation of O1‐defected phases during charging. At the end of charging in LiNiO_2_, the O1 phase is typically observed, which leads to dislocations between the O1 and O3 phases and significant localized oxygen loss near defects.^[^ ^18^ ^]^ Furthermore, once the O1 phase forms within particles, it does not reversibly transform back to the O3 phase. However, in the LiF50, the mitigation of the O1 defect formation helps to retain cycle stability compared to the LiF0. Furthermore, the suppression of the H2–H3 phase transition and the limited lithium kinetics during discharge in LiF50 lead to reduced structural changes (with capacity loss), which also contribute to the improved cycling stability.

### 2.4 Discussion

#### 2.4.1 Formation Mechanism of the Disordering Surface Layer

**Figure 4:**
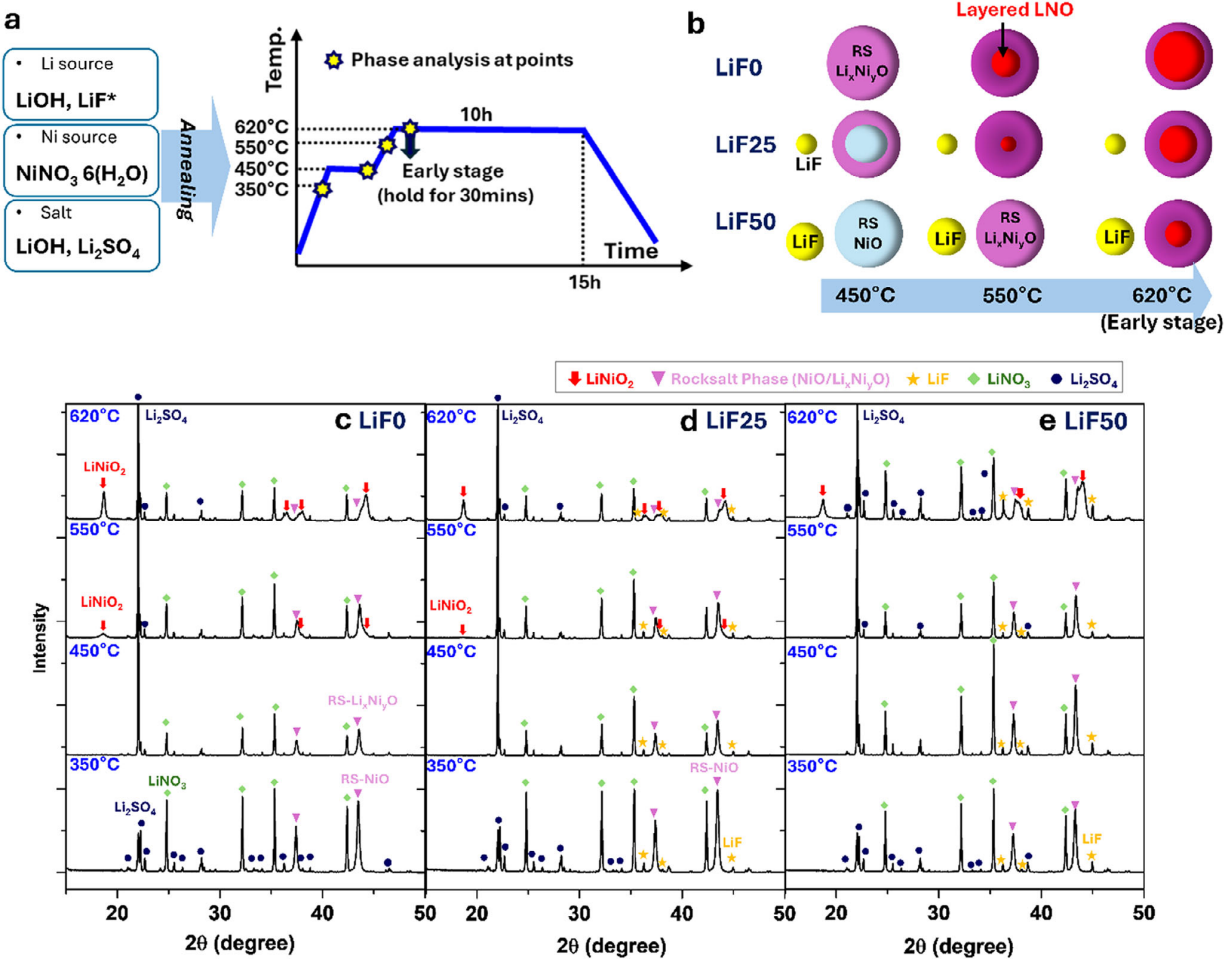
Investigation of the synthesis pathway: a) Schematic representation of the synthesis annealing process, highlighting the intermediate points analyzed XRD patterns. b) A simplified synthesis pathway summarizing the key findings, as derived from the XRD results. Ex situ XRD patterns of the intermediate samples at 350, 450, 550, and 620 °C for c) LiF0, d) LiF25, and e) LiF50.

The disordering phase on the surface is typically observed in Ni‐rich layered cathodes. However, until now, controlling the surface layer during synthesis has not been systematically explored. This work provides insights into a potential method to control the disordered surface layer during synthesis by investigating its formation mechanism. To understand the mechanism, the synthesis pathway was analyzed by examining the XRD patterns of intermediate samples during synthesis at 350, 450, 550, and 620 °C, as depicted in **Figure** **4**a. At 350 °C, the LiF0 exhibited Li_2_SO_4_, LiNO_3_, and rock‐salt (RS)‐NiO. Since NiNO_3_∙6H_2_O was used as a nickel precursor, it appears that decomposed NO_3_ from nickel reacts with lithium to form LiNO_3_ forms at 350 °C, as shown in Figure 4c–e. Interestingly, when LiF is included as a lithium source for LiF25 and LiF50 samples, LiF remains unreacted with NO_3_ due to the strong ionic bonding between lithium and fluorine, as shown in Figure 4d,e.

In the LiF0 sample, as the annealing temperature increased to 450 °C, it is likely that RS‐NiO began transforming into lithiated RS‐Li_x_NiO_y_ through reactions with lithium and oxygen. This is evidenced by the reduced intensities of both RS‐NiO and LiNO_3_ peaks compared to those at 350 °C even though the temperature increased. A similar trend was observed in the LiF25. However, a certain amount of RS‐NiO seems to be remain in LiF25 at 450 °C because the intensity reduction of LiNO_3_ peak was not as much as the LiF0, as shown in Figure 4d. In the LiF50 sample, the intensities of both LiNO_3_ and NiO peaks at 450 °C remained unchanged from those at 350 °C (Figure 4e). Thus, RS‐NiO is likely to be remain at 450 °C in LiF50. It is summarized in Figure 4b.

As the temperature increased to 550 °C, a small intensity of the layered structure, LiNiO_2_ appeared in the LiF0 and the LiF25, with the peaks in the LiF25 being weaker than those in LiF0. Conversely, no LiNiO_2_ peak were observed in the LiF50 at 550 °C, indicating that LiF delays the formation of the layered LiNiO_2_ structure. This delay is likely attributed to the high decomposition temperature and strong bonding of LiF, which limits the availability of lithium for reaction with the RS‐NiO to form the layered structure. Due to the delayed reaction caused by LiF, a thicker rock‐salt phase may remain on the particle surface, preventing complete transformation to the layered structure, as illustrated in Figure 4b. Therefore, the disordered surface layer can be formed or controlled by delaying the reaction that provides lithium to the rock‐salt phase. This delayed reaction, induced by LiF, offers a potential pathway to manipulate the thickness and properties of the disordered surface layer on layered structure particles.

#### 2.4.2 Impact of Crystalline Surface Coating

**Figure 5:**
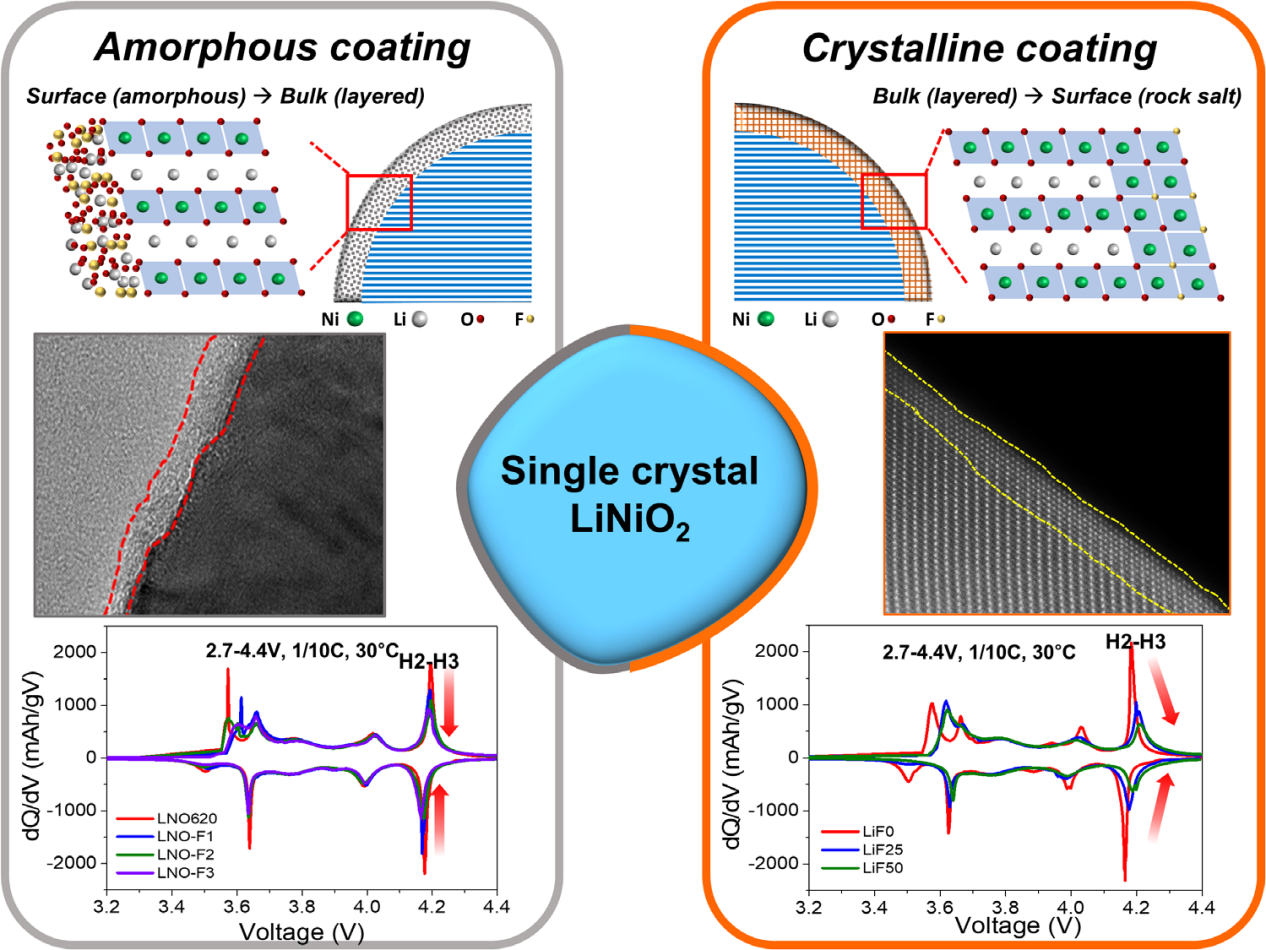
Schematic diagram of surface layer characteristics on single‐crystal LiNiO_2_. Illustration comparing crystalline and amorphous coatings on single‐crystal LiNiO_2_, focusing on their influence on H2‐H3 phase transitions.

Generally, surface coatings conduct on the second annealing step after the synthesis of the cathode at the first annealing. Also, the coating annealing step is performed at a lower temperature than the first annealing with a short time, which aim to prevent bulk structural changes. Due to the process, an amorphous surface coating layer generally forms. Despite the lack of crystallinity, most surface coatings effectively protect the underlying material. In our previous study,^[^ ^12a^ ^]^ the amorphous‐LiF layer was formed on the surface of the same single‐crystal LiNiO_2_, as shown in **Figures** **5** and S8 (Supporting Information), which improved cycle stability effectively as well. In this study, LiF is introduced during the synthesis process replacing LiOH as the lithium precursor, rather than being applied as a coating in a secondary annealing step. During this synthesis process, fluorine segregates on the surface as the LiNiO_2_ phase forms, resulting in the formation of the crystalline disordered phase on the surface coherently. Interestingly, this crystalline surface layer suppresses the H2‐H3 phase transition, a behavior not observed with an amorphous surface coating, as shown in Figure 5. Although amorphous coatings effectively protect the surface, albeit with some capacity loss, they do not influence the reaction voltages associated with the H2‐H3 phase transition (marked by red arrows in Figure 5), regardless of the coating thickness. In contrast, a crystalline surface layer actively suppresses phase transitions, particularly in the H2‐H3 region, which is associated with significant volume changes. Furthermore, this suppression is enhanced with a slightly thicker surface layer. Since the disordered crystalline surface layer forms a coherent interface with the layered structure, it is likely that the lattice changes are constrained by this coherent atomic layer, similar to a pinning effect. This suggests that the lattice parameters of the disordered surface phase remain unchanged, thereby constraining the structural changes within the bulk material. As a result, large volume changes are prevented, which is evidenced by the suppression of the H3 phase transition. These findings indicate that a crystalline surface layer can stabilize both the surface and bulk structure, offering a simple and cost‐effective approach to improving the performance of cathode materials.

## 3 Conclusion

In this study, a crystalline surface coating layer was successfully formed and controlled on single‐crystal LiNiO_2_ by adjusting the LiF precursor ratio during synthesis. The presence of LiF delays the formation of the layered structure from the rock salt phase due to its slow decomposition. As a result, remnants of the rock‐salt phase remain on the surface of the layered structure. Furthermore, as the LiF ratio increases, a thicker disordered phase forms on the surface, accompanied by the segregation of fluorine. This thickened disordered surface layer leads to a decrease in capacity, attributed to the unfavorable lithium diffusion characteristics of the rock‐salt phase. Interestingly, the H2‐H3 phase transition voltages became higher and broader in the dQ/dV profiles as the disordered surface layer thickened. In situ XRD analysis revealed that in the LiF50 sample, the *c*‐lattice parameters of the H2 phase shifted closer to those of the H3 phase, which helps reduce the lattice mismatch between the H2 and H3 phases Additionally, this structural adjustment appears to mitigate the irreversible transition to O1 stacking at the end of charge in the LiF50 sample. Consequently, cycle stability was significantly improved in the samples with a disordered surface layer. In particular, the LiF50 sample exhibited the highest stability, retaining 84% of its capacity after 500 cycles in a full‐cell test—a notably high value for LiNiO_2_. This study demonstrates that a crystalline surface coating not only provides surface protection but also influences phase transitions. While it has been widely accepted that surface coatings do not affect the bulk electrochemical properties, this research provides new insight into how a crystalline surface coating can actively control bulk behavior. This study reveals that a ‘crystalline’ surface coating can influence not only surface protection but also the phase transitions. While it has been widely accepted that surface coatings do not impact the bulk's electrochemical properties, this research provides new insight that a ‘crystalline surface coating’ can control bulk behavior.

## 4 Experimental Section

### Materials Synthesis

Single‐crystal LNO was synthesized via a two‐step molten salt method. 1) In the first step, nickel and lithium precursors were mixed at a 1:1 molar ratio along with a mixed salt flux. Nickel precursor was Ni(NO_3_)_2_·6H_2_O. For the lithium precursor, only LiOH was used in the LiF0 sample, whereas LiOH and LiF were combined at molar ratios of 0.25:0.75 and 0.5:0.5 for LiF25 and LiF50, respectively. The salt flux consisted of a LiOH:Li_2_SO_4_ mixture at a 4:1 molar ratio. The total salt mixture was added at a 2:1 molar ratio relative to Ni(NO_3_)_2_·6H_2_O. All powders were thoroughly ground using a mortar and pestle, then preheated at 450 °C for 2 h, followed by calcination at 620 °C for 10 h in an O_2_ atmosphere. In the second step, the obtained product was washed with deionized water to remove residual lithium salts. The washed powder was then annealed at 700 °C for 4 h under oxygen flow to eliminate surface impurities and any residual moisture. No additional lithium source was added during this annealing step.

### Material Characterizations

Neutron powder diffraction (NPD) was performed at room temperature on the high‐resolution diffractometer Echidna^[^ ^19^ ^]^ using neutrons with a wavelength of 1.6236 Å. To characterize the crystal structure of each sample, powder X‐ray diffraction (XRD) analysis was performed using Cu‐Ka radiation (λ = 1.5418 Å, 40 kV, 25 mA), with a step size of 0.0205°. Rietveld analysis of the NPD and XRD data was performed using the Fullprof program and X'Pert HighScore Plus software package to obtain detailed structural information. In situ charge/discharge XRD measurements of LiF0 and LiF50 were performed at a current rate of C/10, within the 2*theta* range of 10°–70°, using a step size of 0.026° and a duration of 0.3 s per step. The morphologies of samples were obtained by scanning electron microscopy (SEM) using an accelerating voltage of 5 kV on a JSM‐7610F, JEOL LTD, Japan. The size distribution of particles was analyzed by utilizing the ImageJ program to measure the particle sized observed in the SEM images. Selected area electron diffraction (SAED) patterns were obtained using a JEOL JEM‐2100F 200 kV field‐emission TEM instrument. The high‐angle annular dark field–scanning transmission electron microscopy (HAADF‐STEM) images were acquired on a Titan^TM^80–300. The electron energy loss spectroscopy (EELS) results were obtained using the same HAADF‐STEM.

### Electrochemical Analysis

The electrochemical performance of all samples was investigated in 2032‐coin type cell with Li metal as the counter electrode. A homogeneous slurry was prepared by grinding and mixing the active materials, carbon (carbon black, acetylene, 50% compressed, 99.9+%), and polyvinylidene fluoride (PVDF) binder in an agate mortar with N‐methyl‐2‐pyrrolideon (NMP) solvent and stirred for 20 min to obtain a slurry. The mass ratio of the components was carefully controlled at 80:15:5. The slurry was uniformly coated on an Al foil as current collectors (to obtain an active material loading of 5–7 mg cm^−2^), dried in an oven for 2h to evaporate the NMP, and vacuum dried at 120 °C overnight. The fabricated cathodes were punched a circular sheet with a diameter of 13 mm. In a controlled Ar gas environment inside a glove box, coin cells (CR2032) were assembled using a circular cathode sheet, a lithium metal anode, and an electrolyte of 1.2 M LiPF_6_ in ethylene carbonate (EC) ‐ ethyl methyl carbonate (EMC) (3:7 v/v) with 2 wt.% vinylene carbonate (VC). The (dis)charging of the coin cell was conducted using a NEWARE BTS test system within a voltage range of 2.7–4.7 V at charge and discharge rates of 0.5 and 1 C (1 C = 200 mAg^−1^) at 30 °C. Galvanostatic Intermittent Titration Technique (GITT) measurements were carried out using a low current of 0.02 C to investigate the kinetics of lithium ion. Each current pulse lasted for 1 h and was followed by a resting period of 2 h. The voltage range during the measurements was set between 2.7 and 4.4 V.

## Conflict of Interest

The authors declare no conflict of interest.

## Supporting information



Supporting Information

## Data Availability

The data that support the findings of this study are available from the corresponding author upon reasonable request.
