# Supplementary material for: Enhancing Cycle Stability in LiNiO2 with Phase transition suppression via Crystalline Disordered Surface Layer
Source: Adv Sci (Weinh). 2025 Jul 18;12(38):e03476. doi: 10.1002/advs.202503476 (PMC12520496; doi:10.1002/advs.202503476)
Supplement: Supplementary file 1 — Supporting Information [file ADVS-12-e03476-s001.docx]

**Supporting Information**

**Enhancing Cycle Stability in LiNiO₂ with Phase transition suppression via Crystalline Disordered Surface Layer**

Sooyeon Choi ^#1^, Dong-hee Lee^#1^, Yonghyeon Kwon^1^, Maxim Avdeev^2,3^, Seok Hyun Song^4,5^, Minki Kim^6^, Sehyun Kim^6^, Seung-yong Lee^6,7^, Hyungsub Kim^4,8^, Minkyung Kim*^1^

^1#^ The authors are equally contributed

^1^Department of Electronic Materials Engineering, Kwangwoon University, 60 Gwangun-ro 1-gil, Nowon-gu, Seoul, Republic of Korea 01897, Republic of Korea

^2^Australian Nuclear Science and Technology Organisation, New Illawarra Road, Lucas Heights, New South Wales 2234, Australia

^3^School of Chemistry, The University of Sydney, Sydney 2006, Australia

^4^Neutron Science Division, Korea Atomic Energy Research Institute (KAERI), 111 Daedeok-daero 989 Beon-Gil, Yuseong-gu, Daejeon, 34057, Republic of Korea

^5^Gwangju Clean Energy Research Center, Korea Institute of Energy Research (KIER), 270-25 Samso-ro, Buk-gu, Gwangju, 61003, Republic of Korea

^6^Division of Materials Science and Engineering, Hanyang University, Seoul 04763, Republic of Korea

^7^Department of Battery Engineering, Hanyang University, Seoul 04763, Republic of Korea

^8^Advanced Energy Research Institute, Chungbuk National University, Cheongju, Chungbuk 28644, Republic of Korea

**
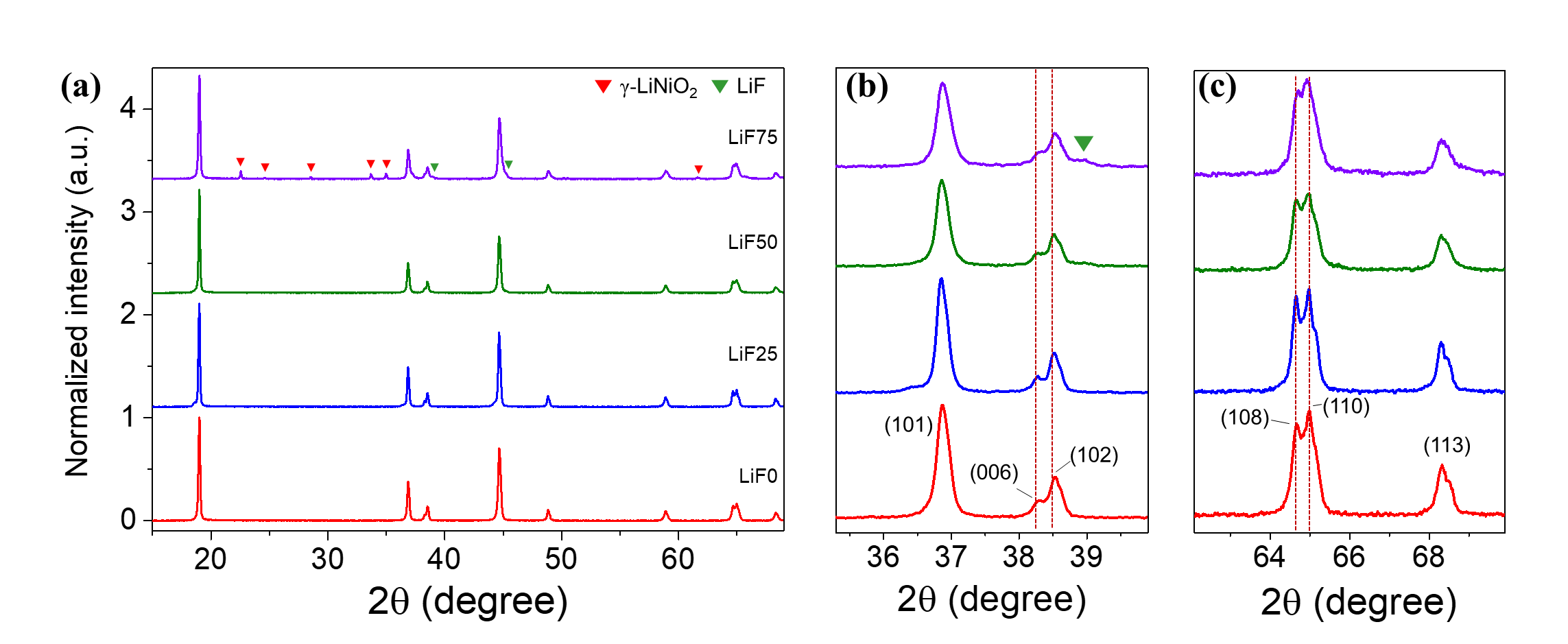
**

**Fig. S1**. (a) XRD patterns of the LiF0, LiF25, LiF50, and LiF75 sample, (b) and (c) Enlarged parts of the XRD patterns.

**Table S1.** Lattice Parameters of LiF0, LiF25, and LiF50 samples by Rietveld refinement of ND patterns.

**
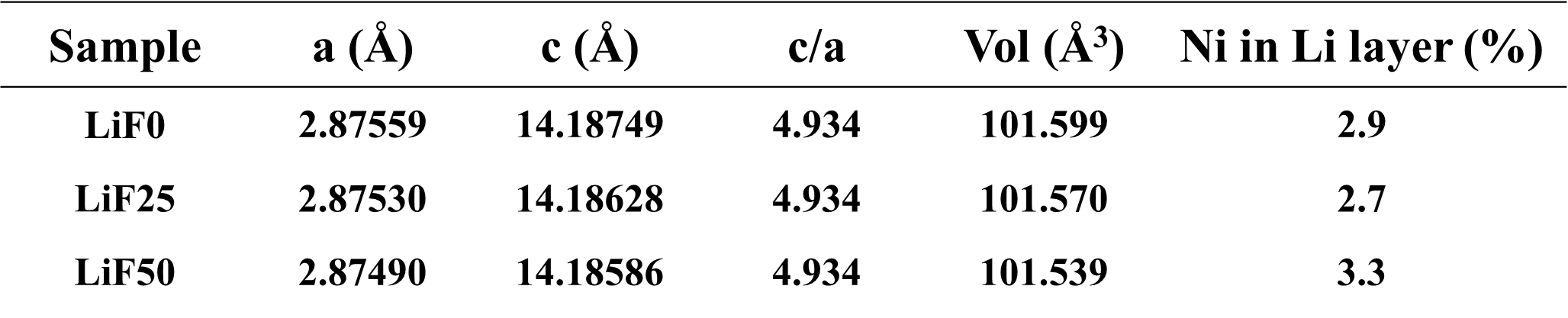
**

**Table S2.** Rietveld refinement results of neutron diffraction patterns.


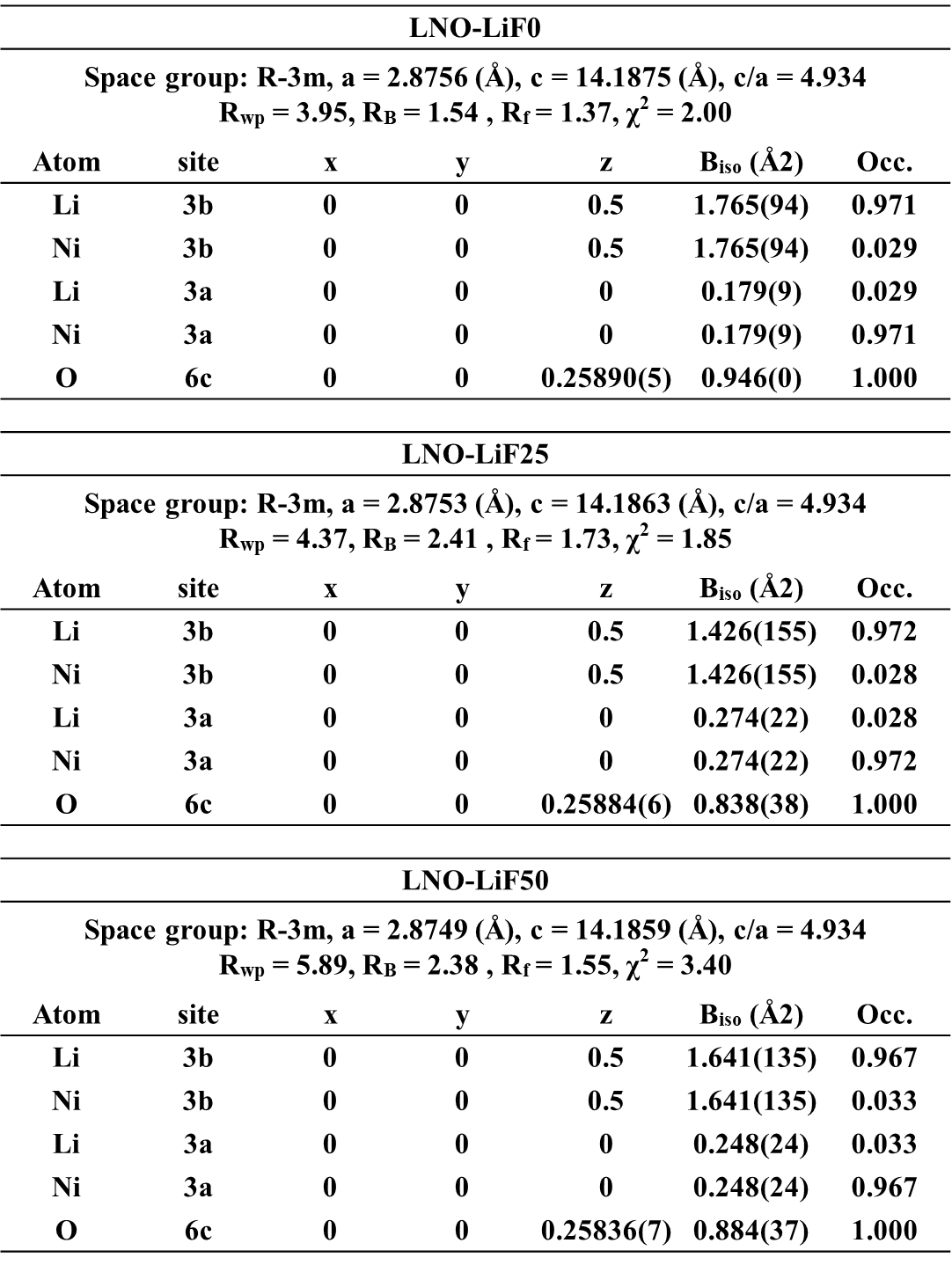


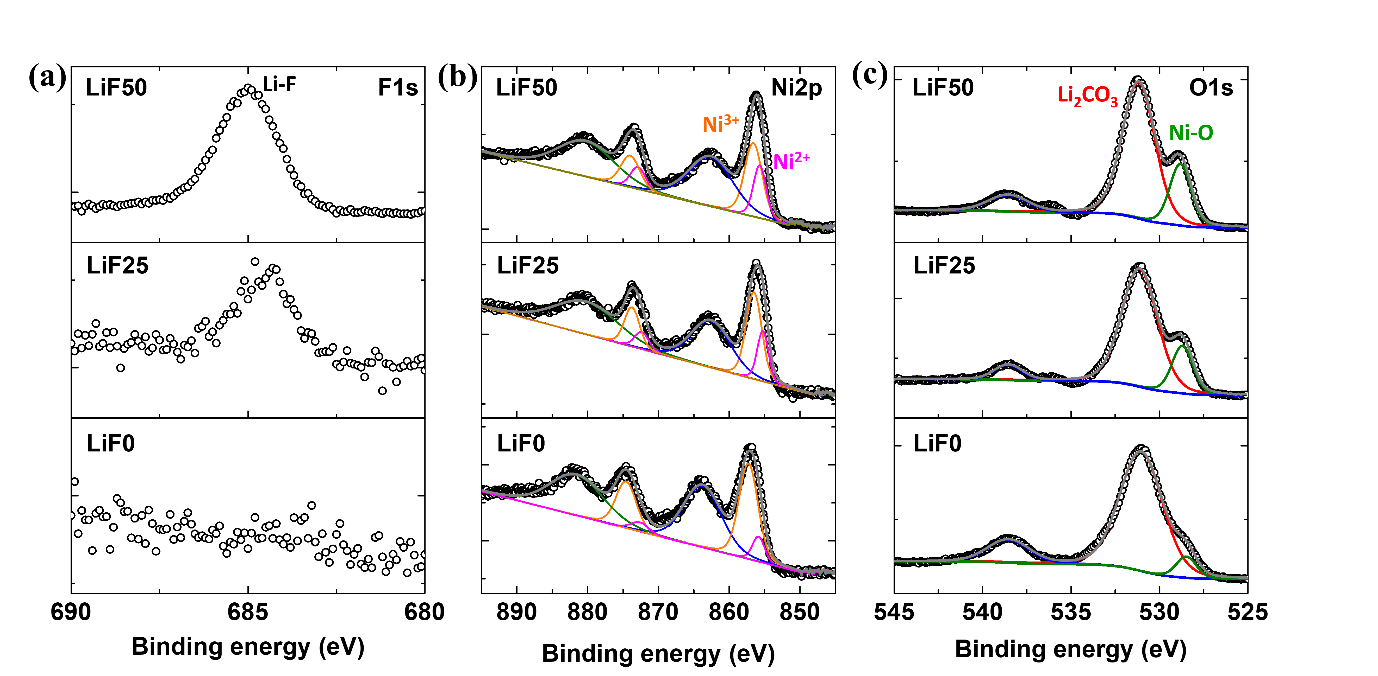


**Fig. S2**. (a) F 1s (b) Ni 2p and (c) O 1s spectra of X-ray Photoelectron Spectroscopy (XPS) compared each sample, LiF0, LiF25 and LiF50.


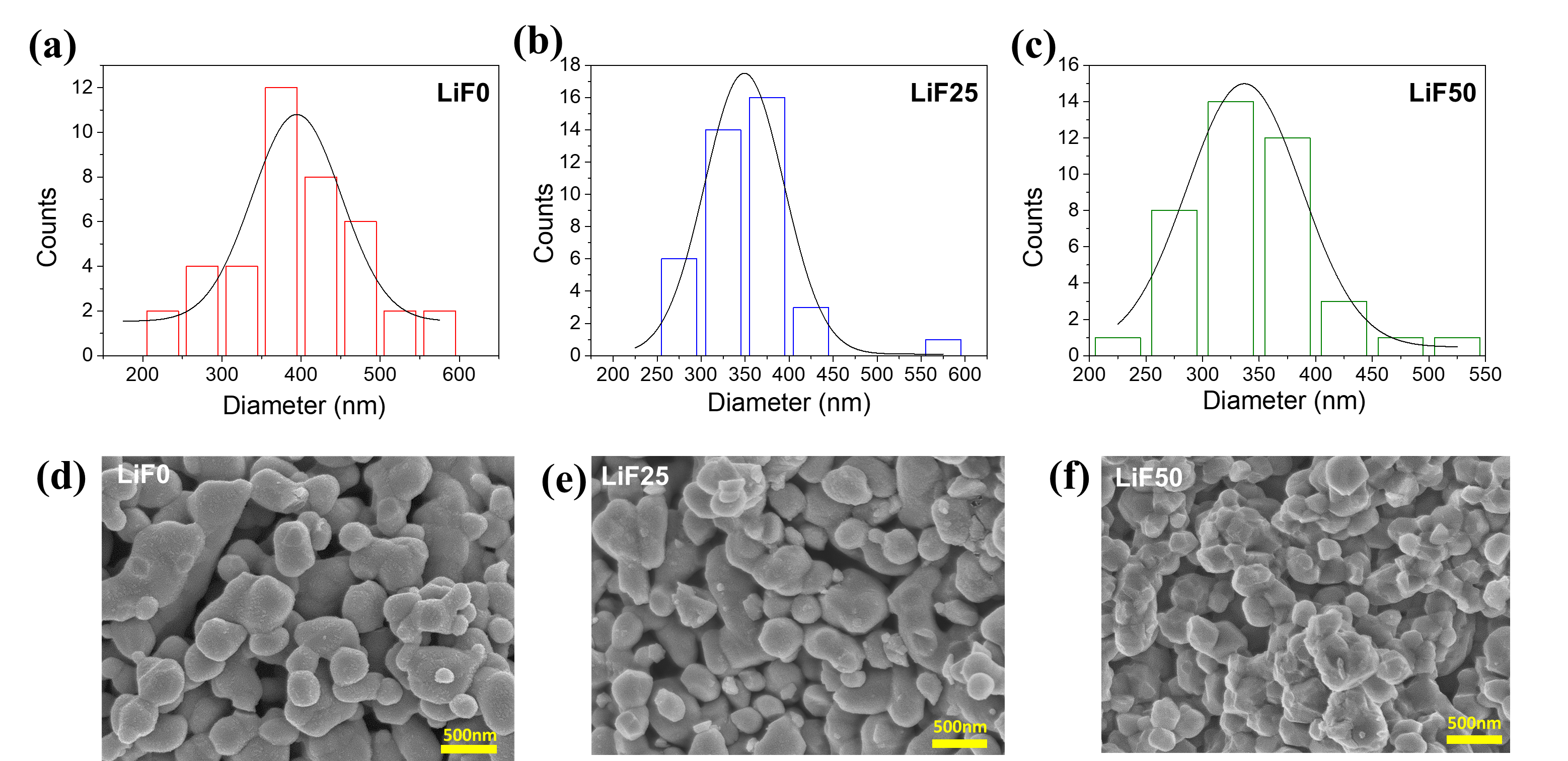


**Fig. S3**. Particle distribution analysis and Scanning electron microscope images of (a), (d) LiF0, (b), (e) LiF25 and (c), (f) LiF50.


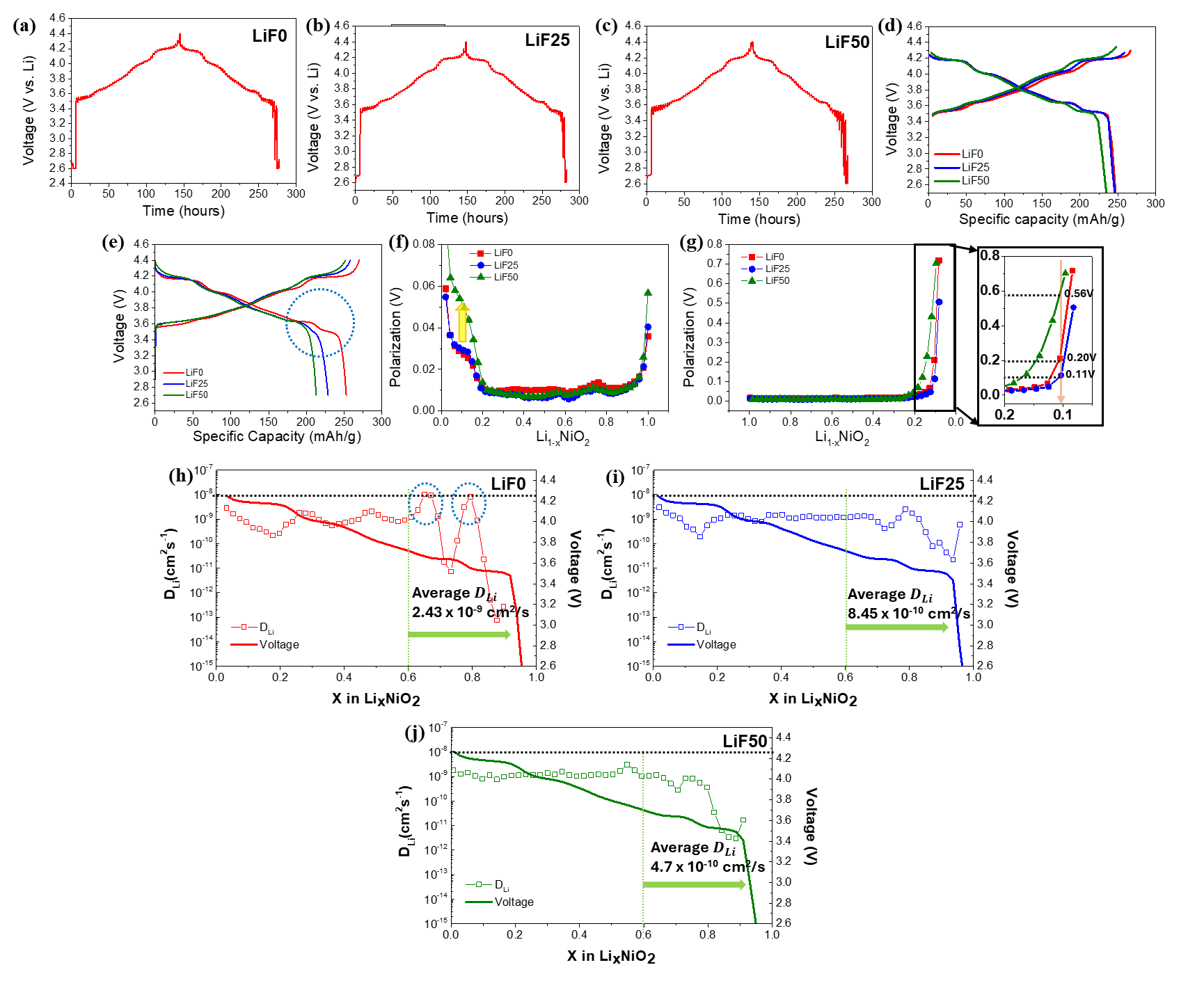


**Fig. S4**. Voltage profile versus time obtained from galvanostatic intermittent titration technique (GITT) for (a) LiF0, (b) LiF25 and (c) LiF50. (d) Quasi-thermodynamic voltage profiles from the GITT test (e) Voltage profiles at C/10 for the samples. Polarization as a function of lithium content during (f) charging and (g) discharging. Calculated lithium diffusivities based on the GITT tests of (h) LiF0, (i) LiF25 and (j) LiF50. Average D_Li_ (x>0.6) presents to kinetics comparison at the end of the region.


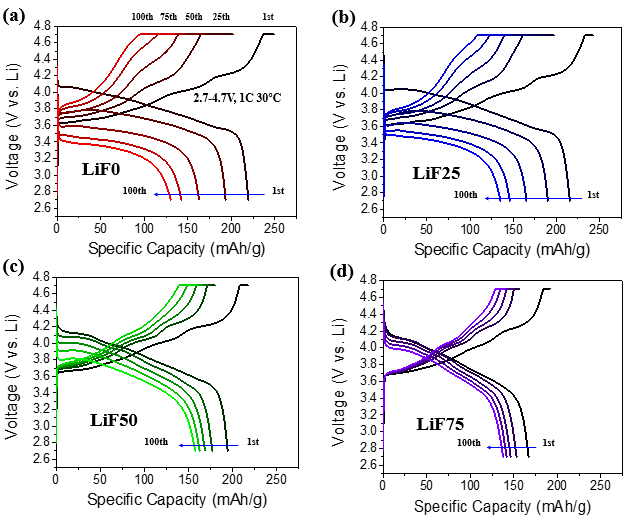


**Fig. S5**. Electrochemical performance charge and discharge curves for different cycles at 1 C of (a) LiF0, (b) LiF25, (c) LiF50, and (d) LiF75 under a hash condition with 4.7 V cutoff.


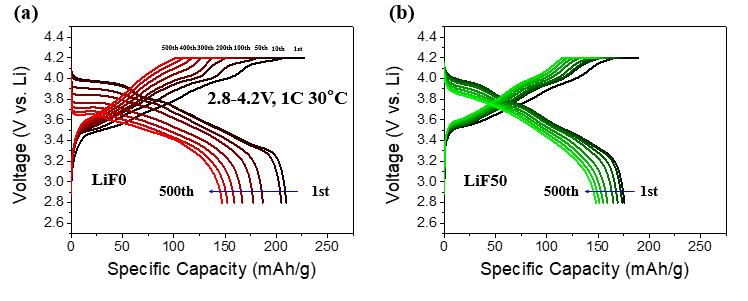


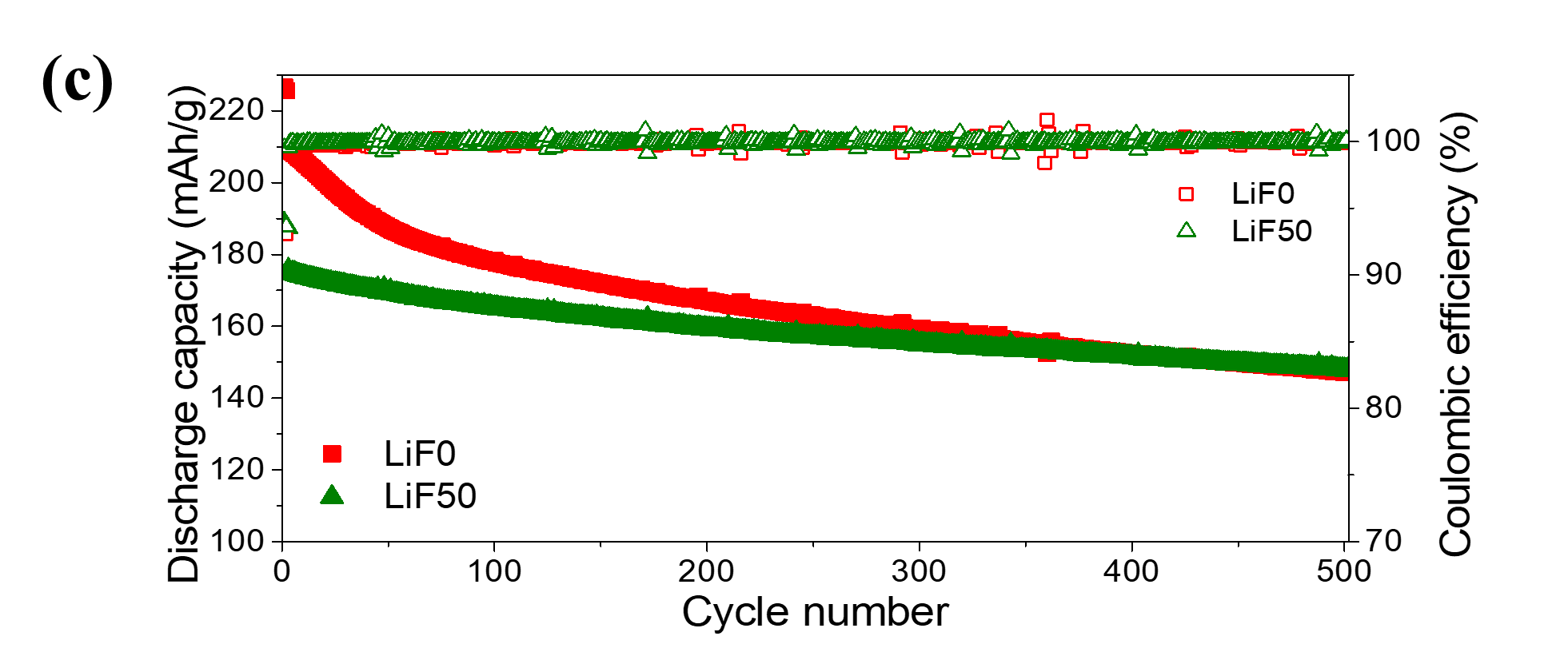


**Fig. S6**. Electrochemical performance charge and discharge curves for different cycles in the full-cell test at 0.8C charging and 1C discharging of (a) LiF0 and (b) LiF50, and (c) capacity retention for 500 cycles.


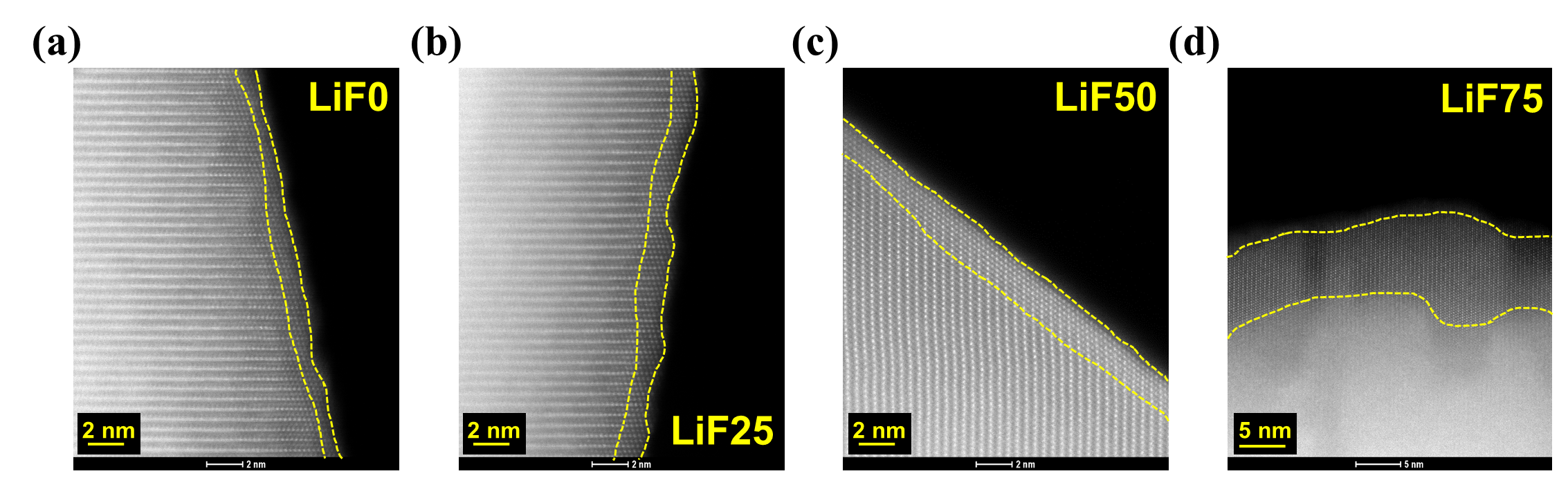


**Fig. S7**. STEM images of LiNiO_2_ surface with different LiF content ratio (a) LiF0, (b) LiF25, (c) LiF50 and (d) LiF75.


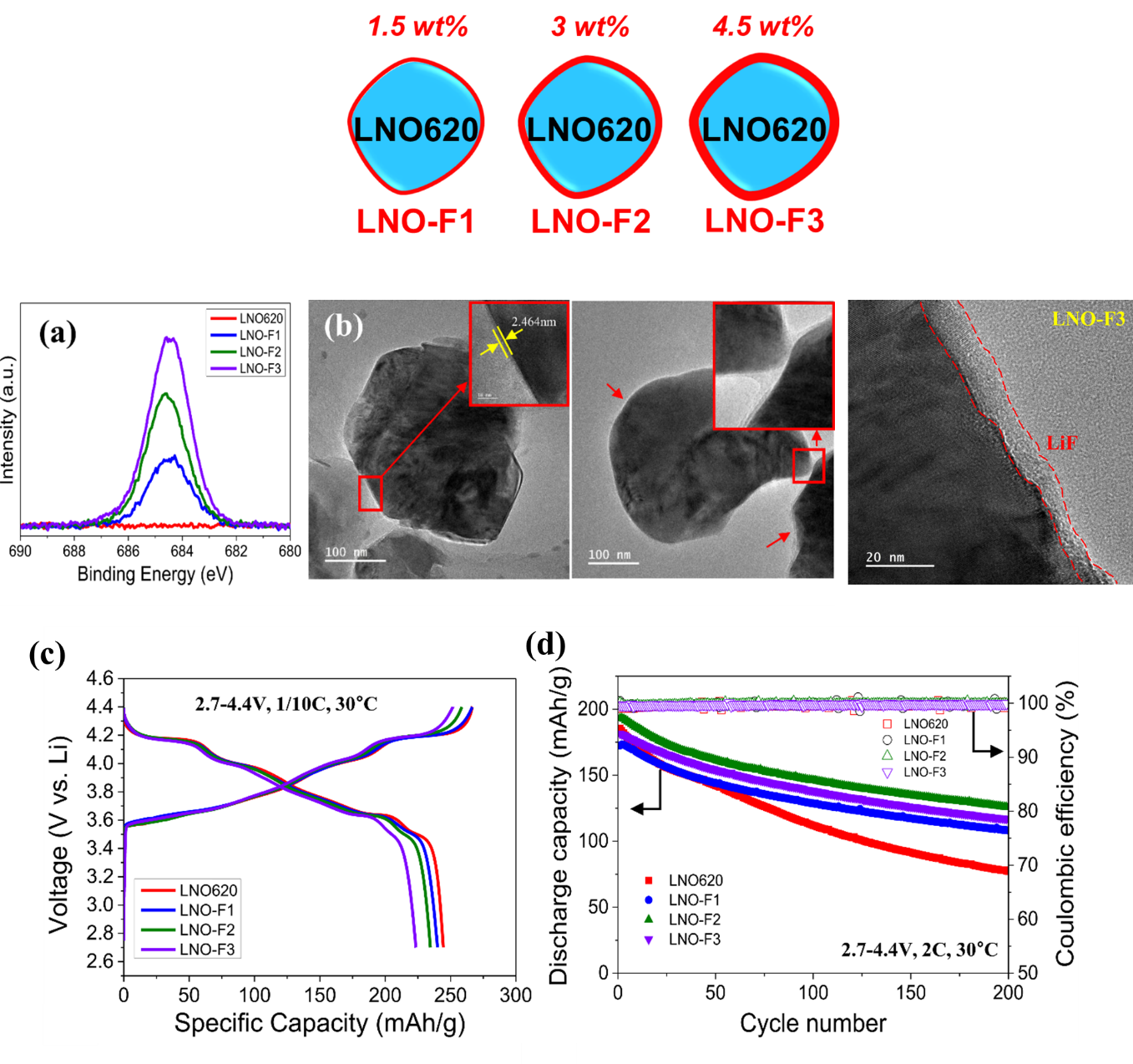


**Fig. S8**. Our previous investigation; amorphous LiF surface coating on the same single crystal LiNiO_2_. LiF coating content varies. (a) XPS spectra F1s (b) TEM images of LNO-F3 (c) Voltage profiles of the samples at 0.1C (d) cycling stabilities of the samples. (Ref: *ACS Appl. Energy Mater.* 2023, 6, 10, 5309–5317)


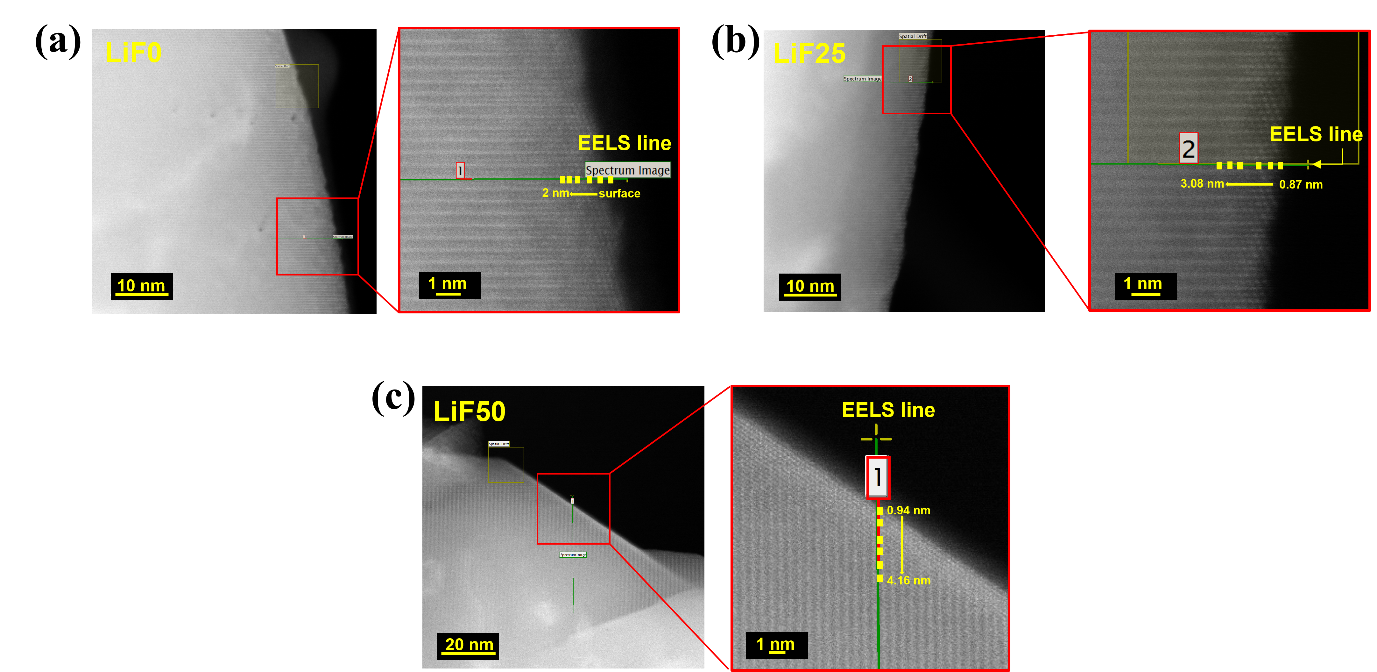


**Fig. S9**. STEM images showing the locations of EELS analysis for the (a) LiF0, (b) LiF25, and (c) LiF50 samples presented in Fig. 1
